# The quest for accountability of Health Facility Governing Committees implementing Direct Health Facility Financing in Tanzania: A supply-side experience

**DOI:** 10.1371/journal.pone.0267708

**Published:** 2022-04-28

**Authors:** Anosisye Mwandulusya Kesale, Christopher Mahonge, Mikidadi Muhanga

**Affiliations:** 1 Department Local Government Management, School of Public Administration and Management, Mzumbe University, Mzumbe University-Morogoro, Morogoro, Tanzania; 2 Department of Policy Planning and Management, Sokoine University of Agriculture, College of Social Sciences and Humanities, Morogoro, Tanzania; 3 Department of the Development Studies-Sokoine University of Agriculture, College of Social Sciences and Humanities, Morogoro, Tanzania; Illawarra Shoalhaven Local Health District, AUSTRALIA

## Abstract

User committees, such as Health Facility Governing Committees, are popular platforms for representing communities and civil society in holding service providers accountable. Fiscal decentralization via various arrangements such as Direct Health Facility Financing is thought to strengthen Health Facility Governing Committees in improving accountability in carrying out the devolved tasks and mandates. The purpose of this study was to analyze the status of accountability of Health Facility Governing Committees in Tanzania under the Direct Health Facility Financing setting as perceived by the supply side. In 32 different health institutions, a cross-sectional design was used to collect both qualitative and quantitative data at one point in time. Data was collected through a closed-ended questionnaire, an in-depth interview, and a Focus Group Discussion. Descriptive statistics, multiple logistic regression, and theme analysis were used to analyze the data. According to the findings, Health Facility Governing Committees’ accountability is 78%. Committees have a high level of accountability in terms of encouraging the community to join community health funds (91.71%), receiving medicines and medical commodities (88.57%), and providing timely health services (84.29%). The health facility governance committee’s responsibility was shown to be substantially connected with the health planning component (p = 0.0048) and the financial management aspect (p = 0.0045). This study found that the fiscal decentralization setting permits Committees to be accountable for carrying out their obligations, resulting in improved health service delivery in developing nations.

## Introduction

Accountability in the health system is necessary for the people to receive accessible, relevant, and responsive health care. Accountability in health care refers to the obligation of health care practitioners or the community to respond to public inquiries regarding their decisions and activities, which are the basis of their mandate, authority, and legitimacy [1, 2]. As a result, accountability encourages accountability between different levels of the health system, resulting in improved health service delivery. Social accountability is highly recommended as an approach for influencing the responsibility of policymakers and health service providers through community participation to improve accountability in public health programs [3]. Social accountability through community participation in holding service provider into account help to address primary health care challenges such as poor utilization and allocation of resources, unresponsive health service delivery and ineffective and inefficient health system [4–6]. In primary health care facilities, social accountability or community participation is represented by community governance structures known as Health Facility Governing Committees (HFGCs). These HFGCs are created to represent communities, civil societies and other interest groups for voicing up and shaping health service delivery in community interest [7, 8]. The HFGCs has two key functions in primary health care: first, they must hold into account health service providers for health facilities to function properly. Second, through community outreach and co-management of health facilities, HFGCs act as an extension of service providers.

The importance of individual, family, and community participation in the management and implementation of health initiatives has been extensively underlined in both the Alma Ata Declaration of 1978 and the Astana Declaration of 2018. Social accountability through various mechanisms such as HFGCs promises to increase health service providers and the health system’s overall accountability. As a result, functional HFGCs are critical in primary health care for improving community health service delivery and addressing the health problems of all individuals. Strengthening community health care through empowering HFGCs means tackling individual and community health concerns by giving them autonomy and the authority to regulate and govern their own health. Universal Health Coverage (UHC) is primarily achieved by ensuring that everyone, including patients and the poor, has access to care, that the care is of sufficient quality, and that no financial obstacles prevent anyone from getting health services. As a result, responsible primary health care is intended to promote population health through well-managed and accountable primary health care facilities that improve population access to health services and quality care while lowering financial obstacles.

Accountability is related to responsibility and responsiveness in a broader sense since it is based on the notion of responding to or being able to complete the given tasks [9]. It is all about account giving or one’s obligation to justify and explain his/her conduct [10]. There are three components of accountability namely the locus of accountability (who), the domains of accountability (what) and the procedure of accountability (how). In primary health care, the locus of accountability refers to who is held accountable or who holds others accountable; in primary health care, this can include nurses, incharges, patients, communities, or community governance bodies like HFGCs [11–13]. The domain of accountability refers to the activity or delegated functions for which a person or entity can be held liable and hence must defend its conduct [9, 11, 14]. The final component is procedural accountability, which refers to the methods that are used to assess a party’s accountability [8]. These can include formal or informal assessments of the locus of accountability’s compliance with the delegated functions, as well as justifications from the accountable part, such as HFGC, to the extent that they have completed their assigned duties [15, 16]. After evaluation, the evaluator can decide to sanction or reward the part held into account.

The interaction between communities and HFGCs in primary health care institutions is best explained using Principal-Agency Theory. The Principal-Agency Theory describes the act of a principle attempting to maximize value/output by engaging/delegating tasks to agents, with the principal regularly monitoring or holding the agents or the agents themselves to account based on their performance [9, 15, 17, 18]. The Principal/Agent Theory marches with the accountability definition that entails the “relationship between an actor and a forum, in which the actor has an obligation to explain and to justify his or her conduct, the forum can pose questions and pass judgment, and the actor may face consequences” [9]. Communities, civil societies, and other interest groups are the primary/forum in which they have assigned their responsibilities to control primary health care facilities through the HFGCs [8, 19, 20]. HFGCs, on the other hand, are agents/actors who are democratically elected by the principal or forum, which is a community or a group of interest groups. As a result, the HFGCs should provide consistent accounting to their electorates, which are communities, whether formally or informally [21]. As suggested by Bovens (9) Three critical aspects must exist between HFGCs (actors or agents) and communities (forum or principal). In the course of carrying out their delegated functions and powers, HFGCs (agents) are required to inform communities and other interest groups (principals) about their actions. Similarly, communities and other interest groups (principals) could question the HFGCs (agent/forums) about many aspects and information relevant to health service delivery in their communities or health institutions. Finally, after hearing the HFGCs’ responses, the communities and civil societies represented by HFGCS may be able to cast judgment on the HFGCs. The verdict may be favorable if communities and civil societies believe that HFGCs are performing well, but citizens may impose sanctions if they believe that HFGCs have failed to carry out their duties and authorities. As a result, justifying, explaining, reporting, and disciplining may all be considered accountability [9].

Despite the fact that the global health community recognizes the HFGCs’ importance in overseeing the execution of primary health care plans, there is limited evidence about HFGC accountability in achieving social accountability under fiscal decentralization [6, 9]. The available empirical evidence has been devoted to investigating the method utilized by HFGCs to hold healthcare providers accountable [6, 7, 21], the link between management competency, accountability, and hospital board governance [10]. Furthermore, studies have shown the linkage between citizens and elected politicians [15]. Lodenstein et al. [5] discovered that the HFGC accountability cycle is less practiced and institutionalized in Sub-Saharan African countries. Several low- and middle-income countries are now delegating budgetary authority and responsibility to HFGCs.

### The Direct Health Facility Financing context in Tanzania

HFGC was established at health centers and dispensaries in Tanzania as part of the Health Sector Reforms in 1999 to represent communities in the management of health services offered in health facilities. The 2013 guidelines of the Council Health Service Board (CHSB) and Health Facility Governing Committees (HFGCs) [22] have assigned HFGCs specific functions. Participating in the mobilization of financial resources, motivating residents to join enhanced community health funds, and preparing health facility plans and budgets are examples of these functions. In addition, managing the facility’s income and expenditure, discussing community health concerns and developing solutions, and assessing community needs and preferences. Participating in the acquisition of medicine and medical commodities, as well as the development and maintenance of health facility infrastructure. Several studies were undertaken in Tanzania to analyze the accountability and performance of HFGCs since their establishment, however, revealed that HFGCs were not accountable because they were not carrying out their duties properly. Boex and WHO [23, 24] It was discovered that HFGCs were not properly carrying out their duties and powers because budgetary control and authority over primary health care facilities had been delegated to council levels via Council Health Management Committees (CHMT) rather than HFGCs and health providers. Furthermore, facility monies were placed into council accounts and administered by the CHMT. Health providers and HFGCs have no authority over or direct access to facility money. Tanzania’s government introduced DHFF to address this issue for Health Facilities and HFGCs. DHFF is a Tanzanian government initiative that empowers and grants autonomy to HFGCs and basic health care facilities to plan, budget, and manage facility financing in order to improve health service delivery [19]. It utilizes the term DHFF since payments from various sources are transferred directly to the public primary health facility bank account. This type of fiscal decentralization is commonly used in Tanzania’s public basic health care institutions. The DHFF implementation began in all Tanzanian district councils during the fiscal year 2017/18.

Despite the implementation of fiscal decentration through DHFF in all public primary health care facilities to empower HFGCs with fiscal and decision-making capabilities while overseeing health facility delivery, the status of HFGC accountability in primary health facilities implementing DHFF is unknown. Indeed, there is no agreement or guidelines in place to assess the accountability of HFGCs in the process of managing and implementing health plans and operations in order to improve the quality of health service supply. This study examines the level of HFGC accountability and the factors that influence it in Tanzanian primary health care facilities that are implementing DHFF.

## Materials and methods

### Research design

The study used a cross-sectional design, in which both quantitative and qualitative data were collected at the same time in selected health facilities throughout four regions that have implemented Direct Health Facility Financing.

### Sample size and sampling techniques

This study used both probability and non-probability sampling procedures to select the representative sample from the population [25]. The research units were chosen using a multistage sampling process. The selection was based on a Star Rating Assessment, which was carried out in early 2018, the same year that the DHFF implementation began. The President’s Office of Regional Administration and Local Government completed the star rating assessment in all primary health care facilities in Tanzania. The primary goal of the star rating assessment was to examine the performance of health care facilities and provide feedback for future improvements. One of the topics analyzed in the star rating evaluation report was social accountability (Service Area 8), in which the functionality of HFGCs was evaluated and HFGCs with low and high functionality were identified [26]. The domains utilized to evaluate the functionality of HFGCs were the number of meetings held by the HFGC per year, issues covered at HFGC meetings, HFGC engagement in the planning and budgeting process, and communication between the community and the HFGC. Other factors were concerns discussed and resolved at HFGC meetings, HFGC orientation and training, and HFGC engagement in the procurement process. The sampling procedure is summarized in Table 1.

**Table 1:** Sampling process and sampling techniques.

Stage	Respondent	Sampling procedure	Inclusion criteria
First stage	Four (4) regions selected Kilimanjaro, Mbeya, Ruvuma and Songwe	• purposive	Performance of the region, Zonal representation
Second Stage	8 LGAs selected; Two LGAs from each region were selected in stage one	• purposive	Performance of the LGAs in star rating assessment, nature of the LGA (Urban and Rural)
Stage Three	32 health facilities were selected from all (8) councils. 2 health centers and 2 dispensaries from each LGA because they all implement DHFF	• Stratification of health facilities into Health centers and Dispensaries • Purposive selection of health centers and dispensaries	Performance of health facility (A good and poor performing health center and dispensary), Location of the facility within the LGA (Diversity)
Stage Four	280 HFGC members; 9 members from each selected health facility	• Simple random selection of HFGC members	members of the HFGC

#### Quantitative sample size

At stage four, the HFGC representatives were obtained using the proportion sampling technique suggested by [27, 28], The formula assumes a 95% confidence level and a P of 0.5. As a result of the strategies, the number of HFGCs members required was 288. The number of HFGC members from each selected health facility was then estimated using the proportional sampling technique developed by [29] where 9 HFGC members were meant to be chosen from each HFGC For this study, the total number of simple size respondents (response) from all health facilities was 280.

#### Qualitative recruitment of participants

Purposive qualitative recruitment was carried out. The participants were chosen for interviews and focus groups based on their capacity to provide meaningful information about the accountability under HFGCs under Direct Health Facility Financing. Chairpersons of HFGCs were purposely chosen for interviews because they expected to be well-versed in the functionality and responsibility of HFGCs. In the case of FGDs, all members of HFGCs were involved because they were all expected to assist in carrying out HFGC’s duties and obligations. The point of saturation determined the amount of 14 interviews and 16 focus groups. Saturation occurred when interviewers and FGD participants continued to provide similar responses, resulting in no new information being supplied throughout the interview. Because qualitative participants were a subset of quantitative participants, their profiles are comparable to those of the quantitative participants. The HFGC chairperson and members were involved in the quantitative collection.

### Data collection methods and techniques

#### Quantitative data collection

A systematic closed-ended questionnaire was used. Face-to-face interviews with participants were utilized to obtain data from selected HFGC members. The Open Data Kit software was used to develop the data gathering software (database) (ODK). The acquired data was then entered into the ODK. To collect data, a quantitative approach based on mobile data collecting (MDC) was used. After data was captured via mobile phones, it was transferred to a central server. Four research assistants who were interviewing respondents received three days of training on mobile data collection skills and methodologies, followed by pre-testing of the imparted skills at selected facilities outside the study region. The acquired data were provided to the researcher using the ODK platform. All selected facilities had GPS coordinates as part of quality control, so all research assistants used tablets with GPS sensors. The response rate for HFGCs who completed the questionnaire was 280 out of 288.

#### Qualitative data collection

In-depth interviews with HFGC chairpersons were undertaken to examine the group’s responsibility. The interview guide, which included an accountability index, was used to question the HFGC chairpersons. To acquire qualitative data, a Focus Group Discussion (FGD) involving HFGC members was also used. In the health facilities chosen for this study, interviews and focus groups were held.

#### Data collection tools

Based on the delegated tasks of HFGCs as allocated by the HFGC establishing guidelines and DHFF protocol in Tanzania, quantitative data collection techniques were created into an accountability index. This study did, in fact, use the accountability indicators used by the star rating assessment to assess the functionality of HFGCs. As a result, the broader issues that informed the questionnaire development were financial management, planning, and budgeting, community linkages and complaints, and community mobilization to join enhanced community health insurance. Others were involved in procurement, performance management, and service quality assurance.

We generated qualitative data collecting guides based on the HFGC functions allocated to HFGCs in Tanzania by the HFGC guideline of 2013 and DHFFprotocol, which correlate to the indicators used to assess the functionality of HFGCs during the 2018 Star Rating Assessment.

### Data analysis

To determine if HFGCs act to fulfill their tasks, descriptive statistics were used to assess their responsibility. The accountability of HFGCs was assessed using predictors of accountability such as the availability of a price list, a suggestion box, meeting minutes, and evidence of communication between the HFGC and the community. Interviews and FDGs were transcribed verbatim for qualitative data analysis. In-depth interviews took an average of 25 minutes, while focus groups took an average of 32 minutes. The analysis of the audio data began with defining or selecting elements of the recorded audio that were connected to the HFGCs’ accountability index. Multiple Logistic Regression was employed to assess the factors associated with the accountability of HFGCs. The selected parts of the audio-recorded interview and FDGs were then transcribed. The transcription of the audio was completed by the Research Assistant who was in charge of gathering it. The response of the participants was evaluated deductively using the direction of Principal agency theory after transcription of the text statement demonstrating the feelings and experience of the HFGCs in carrying out their duties on the implementation of DHFF. As a result, the statement referring to the experience of HFGC members’ participation in various HFGC functions was reviewed to assess accountability.

#### Data cleaning

The data cleaning was done especially for open-ended questions like transportation used to reach the Health facility, which allowed research assistance to write, some of them wrote "by car" and others wrote "Car" because they have the same meaning we renamed car to by car because they have the same meaning

The random missing did not detect because the data was collected using a mobile device via the ODK platform, there were data quality checks on a daily basis for any observation, and we communicated with research support for explanation and direction.

#### Ethical approval and informed consent

Sokoine University of Agriculture provided ethical approval or an IRB for the project. The Sokoine University of Agriculture provided the IRB with the number SUA/ADM/R. 1/8/668. The permit was then filed to the President’s Office of Regional Administration and Local Government (PO-RALG) to be granted permission to conduct research on local government authorities. PO-RALG granted the researcher a permit with the registration number AB.307/323/01 to conduct research in the chosen areas. All human participants in this study gave their informed permission. Those who agreed to participate in the study and signed informed consent papers before doing so.

The study, however, was subject to various biases, such as the giving of monetary incentives to the participants. Face masks, sanitizers, and transportation allowances were among the incentives provided. Because the data was collected during the second wave of COVID 19 in Tanzania from February to April 2021, face masks and sanitizers were provided. As a result, adherence to the COVID 19 protocol was prioritized, despite the fact that this may be perceived as having an impact on participants. Participants who lived a long distance away from the health center where the data was collected were given transportation.

## Results

The demographic profile included 280 respondents from four regions who were members of the HFGCs. Respondents were classified according to the type of health facility, their position in the HFGC, their age in terms of years, sex, and educational level, as indicated in Table 2 below.

**Table 2:** Demographic characteristics of HFGs members N = 280.

Variable	Frequency	Percent
**Region**		
Kilimanjaro	93	33.21
Mbeya	64	22.86
Songwe	54	19.29
Ruvuma	69	24.64
** Type of Health Facility**		
Dispensary	161	57.50
Health center	119	42.50
** Position**		
Chairperson	43	15.36
Secretary or facility in charge	34	12.14
Member of the HFGC	203	72.50
** Age**		
<30	32	11.43
31–45	100	35.71
46–60	107	38.21
61+	41	14.64
** Sex**		
Male	139	49.64
Female	141	50.36
** Education level**		
Primary	150	53.57
Secondary	64	22.86
Certificate	24	8.57
Diploma	30	10.71
Advanced diploma	5	1.79
University degree	7	2.50

### HFGCs accountability index

The developed accountability index of the HFGC in developing nations is shown in Table 3 below. This accountability index was produced based on a review of the literature, the Tanzania HFGC guideline, and the DHFF protocol, which illustrate the functions that HFGCs are required to execute in the course of governing and managing health facilities.

**Table 3:** HFGCs accountability index.

HFGC Accountability Index
Linkages with stakeholders to identify health challenges
Established collaboration with other development partners
Convened HFGCs official meetings as per schedule
Presented and discussed facility plan implementation reports in HFGCmeetings
Evidence on the matching of facility resources with patients or community needs
Timely care to facility patients when they attend a health facility
presented to the Ward Development Committee/ Village Council
Authorized funds by HFGC as per budget
Facility expenditure did as per financial guidelines
Discussed quarterly facility financial reports in HFGCs quarterly meetings
Participation of HFGC in the facility procurement process
Participation of HFGC in the planning and budgeting process
Participation of HFGC in receiving medicines and other goods
HFGC participation in staff motivation, recruitment and training
HFGC ensures income and expenditure are known to the community quarterly
HFGC ensures the suggestion box is available in a location where it can be seen by the patients
HFGC ensures the price list for services provided is displayed to the extent that can be seen by the patients
HFGC participates in mobilizing the community to join improved community health funds
HFGC ensure the Mobile number and names for complaints are displayed in a location where they can easily be seen by users
HFGC ensures the client service charter of the facility is displayed in a location where it can easily be seen and read by the health service users

### Accountability of HFGCs

To assess the accountability of HFGCs in Tanzania, prepared accountability indexes were distributed to respondents in order to determine the extent to which their HFGCs have met all of the aspects of accountability. Respondents were asked to rate the extent to which the HFGC achieves that data in each item. The information is presented in Table 4 below.

**Table 4:** Perceived accountability of HFGCs in the public primary health facilities implementing DHFF in Tanzania N = 280.

Statement on the extent HFGC accomplishes their Responsibilities	High Acc N (%)	Low Acc N (%)
HFGC communicates with other stakeholders to identify health challenges and needs	150(53.57)	130(46.43)
HFGC has established collaboration with other development partners to work together in providing services to the community	201(71.79)	79(28.21)
HFGC convene meeting with Facility Health workers to discuss different issues of our facility	222(79.29)	58(20.71)
HFGC ensures Health facility progressive reports are presented in the HFGCs meetings	227(81.07)	53(18.93)
HFGC ensures that health facility resources match patient’s or Community needs	214(76.43)	66(23.57)
Patients receive timely care when they attend our health facility	236(84.29)	44(15.71)
Facility progressive reports are presented to the Ward Development Committee/ Village Council	224(80.00)	56(20.00)
HFGC authorizes the use of funds as budgeted	230(82.14)	50(17.86)
HFGC ensures facility funds are used as per financial guidelines	229(81.79)	51(18.21)
HFGC ensures financial reports are provided quarterly and comply with the reporting systems	227(81.07)	53(18.93)
HFGC endorses and participates in the procurement process of all goods and services of the health facility	225(80.36)	55(19.64)
HFGC participates in the planning and budgeting process	229(81.79)	51(18.21)
HFGC participates in receiving medicines and goods procured by our facility	248(88.57)	32(11.43)
HFGC d make a recommendation on staff motivation, recruitment and training to the Council Health Service Board	122(43.57)	158(56.43)
HFGC ensures income and expenditure are known to the community quarterly	188(67.14)	92(32.86)
The suggestion box is available in a location where it can be seen by the patients	203(72.50)	77(27.50)
The price list for services provided is displayed to the extent that can be seen by the patients	192(68.57)	88(31.43)
HFGC participates in mobilizing the community to join improved community health funds	254(90.71)	26(9.29)
he Mobile number and names for complaints are displayed in the location where they can easily be seen by users	214(76.43)	66(23.57)
The client service charter of the facility is displayed on the location where it can easily be seen and read by the health service users	176(62.86)	104(37.14)
**Overall accountability**	**220(78.57)**	**60(21.43)**

Table 4 shows the results of HFGC members’ perceptions of the HFGC’s accountability at public primary health institutions implementing DHFF in selected Councils in Tanzania. In general, the results show that HFGC members view HFGCs to have high accountability for 78.57 percent of the time and low accountability for 21.43 percent of the time. Specifically, it is perceived that HFGCs are more or have higher accountability in mobilizing communities to join Improved Community Health Funds, receiving medicine, medical commodities, and goods, ensuring patients receive timely care in their facilities and authorizing funds per the budget. Meanwhile, HFGCs have been found to have low accountability on topics such as employee motivation, recruiting, and training, engaging with stakeholders to identify health challenges, and ensuring the client services charter is applied successfully in health facilities.

### Experience of HFGCs on their accountability in primary health facilities implementing DHFF

Participants responded to several themes during FGDs and in-depth interviews, but the themes that emerged as common among respondents were mobilizing communities to join Improved community health funds, participating in Receiving medicine and medical commodities, financial management (authorizing expenditure and income), and collecting and discussing community health challenges.

#### Financial management

Participants’ responses on how they have been fulfilling their obligations of managing financial resources varied in this theme area.

*"We constantly review financial condition because without finances*, *you can’t manage the facility*, *therefore finance was number one*, *how to boost revenue*, *and how to spend it*.*"* (FGD 15-High performing Facility, Chunya District Council)

Another participant responded

*"I believe the agenda that is unavoidable in the meeting when we meet is about how much we have collected (revenue collection and future plans)*.*"* HFGC Chairperson- High performing facility, Mbeya City Council)

*"In financial management*, *we make sure that all funds are deposited into the facility bank account*, *and if there is a need for funds*, *such as paying the cleaners*, *we convene a committee meeting and agree on the transaction*.*"* (FGD 1-Low performing facility, Madaba District Council)

#### Mobilizing community to join community health fund

Participants reacted to the way they have carried out their responsibilities in ensuring community people join enhanced community health funds in the individual primary health facilities through focus groups and in-depth interviews.

*"We organize the community by speaking with patients when they visit the facility*, *and we also speak with the village chairperson to assist us during village meetings so that we can continue mobilizing the community"* (HFGC Chairperson- High Performing facility, Songea Municipal Council)

#### Procuring and receiving medicine and medical commodities

One of the main responsibilities of HFGC committees is to guarantee that they are involved in identifying the medicines and medical supplies that health facilities require. They also endorse medications and medical commodities to be procured by the facility, and they are a part of the team that receives medicines and medical commodities procured according to orders. In terms of how well they perform this function, respondents had the following reactions.

*"We always question the health facility in charge about the availability of medicines and medical supplies*, *and then we negotiate a new structure of receiving them either through prime suppliers or the Medical Commodities Department*,*"* (FGD 11- low-performing Facility-District Council of Chunya)

Another participant added

*"We are part of the medical reception team*, *so we evaluate medicines and medical supplies to verify whether they match what we requested; if they don’t*, *we don’t receive them*.*"* HFGC Chairperson–High performing facility, Moshi Municipal Council)

#### Reporting, collecting and discussing with community about health facility operations and challenges

Participants testified about how they have communicated the progress and plans of their health facilities to the community. They also talked about how they have dealt with community health issues and how they have managed their health care facilities. Participants also agreed that the fundamental function of HFGCs at primary health care facilities is to identify, discuss, and resolve community health concerns. Above all, they acknowledged the importance of the HFGCs members’ existence to the powers of the communities. They testified that they were voted to serve on HFGCs because the community believes they are capable of managing the health facilities. Participants responded in the following ways during focus groups and in-depth interviews:

*"We have communicated to the community what we are doing and the status of the health center operations through meetings with communities and another gathering*.*"* (HFGC Chairperson- Moshi Municipal Council)

*“As members*, *we collect and debate community health challenges*… *When a member of the community lodges a complaint*, *we collaborate with health experts to determine the best method to address it*.*"* (FGD 3- Tunduma Town Council- High-Performance Facility)

Another participant said

*"Some of us here have been in this HFGC for three terms because the community trusts us and has voted for us in every election because they believe we are doing a great job of reforming health service delivery at this institution*.*"* (FGD 2- Mbozi District Council-Low performing facility).

### Factors associated with the accountability of the health facility governance committee

As indicated in the methodology section, binary logistic regression was used to examine parameters related to accountability. The results are shown in Table 5 below. The accountability of the health facility governance committee was found to be substantially related to the health planning element (p = 0.0048) and the financial management component (p = 0.0045). In terms of health planning, the results revealed that health facility governance committees with effective planning were considerably more likely to have high accountability than their counterparts (AOR = 5.46, p = 0.0048). Members of the committee who had good financial management were more likely to have high accountability than those who had bad financial management [(AOR = 5.33, p = 0.0045).

**Table 5:** Binary logistic analysis for factors associated with the accountability of HFGCs.

Variable	High Accountability	Low Accountability	Unadjusted		Adjusted	
	N (%)	N (%)	OR[95%CI]	p-value	OR[95%CI]	p-value
**Type of Health Facility**						
Dispensary	124(77.02)	37(22.98)				
Health center	96(80.67)	23(19.33)	1.25[0.69, 2.24]	0.4619		
**Position**						
Chairperson	35(81.40)	8(18.60)	ref			
Secretary	30(88.24)	4(11.76)	1.71[0.47, 6.26]	0.4148		
Member of the HFGC	155(76.35)	48(23.65)	0.74[0.32, 1.69]	0.4752		
**Age**						
<30	21(65.63)	11(34.38)	ref		ref	
31–45	72(72.00)	28(28.00)	1.35[0.58, 3.15]	0.4923	1.69[0.46, 6.24]	0.9151
46–60	93(86.92)	14(13.08)	3.48[1.37, 8.74]	0.0080	3.13[0.72, 13.59]	0.8366
61+	34(82.93)	7(17.07)	2.54[0.85, 7.59]	0.0939	0.49[0.09, 2.59]	0.6903
**Sex**						
Male	108(77.70)	31(22.30)	ref			
Female	112(79.43)	29(20.57)	1.11[0.63, 1.96]	0.7236		
**Education level**						
Primary	115(76.67)	35(23.33)	ref		Ref	
Secondary	51(79.69)	13(20.31)	1.19[0.58, 2.45]	0.6279	1.06[0.35, 3.22]	0.9151
Certificate	17(70.83)	7(29.17)	0.74[0.28, 1.93]	0.5363	0.86[0.19, 3.75]	0.8366
Diploma or above	37(88.10)	5(11.90)	2.25[0.82, 6.17]	0.1143	1.36[0.29, 6.19]	0.6903
**Governance**						
Poor	25(35.21)	46(64.79)	ref		ref	
Good	195(93.30)	14(6.70)	3.06[1.22, 7.65]	0.0169	1.05[0.26, 4.19]	0.9461
Participation in Health Planning and Budgeting						
Not good	35(41.67)	49(58.33)	ref		ref	
Good	185(94.39)	11(5.61)	25.6[12.4, 53.12]	< .0001	5.46[1.68, 17.77]	**0.0048**
Participation Financial management						
Poor	33(41.25)	47(58.75)	ref		ref	
Good	187(93.50)	13(6.50)	23.55[11.2, 49.7]	< .0001	5.33[1.68, 16.89]	**0.0045**
Partcipation Procurement process						
Poor	56(53.33)	49(46.67)	ref		ref	
Good	164(93.71)	11(6.29)	20.49[10.0, 41.9]	< .0001	2.84[0.85, 9.46]	0.0893
Informational reports						
Poor	114(66.67)	57(33.33)	ref		ref	
Good	106(97.25)	3(2.75)	13.05[6.34, 26.8]	< .0001	1.42[0.43, 4.66]	0.5662
Participation in Human resources management						
Poor	186(76.54)	57(23.46)	ref		ref	
Good	34(91.89)	3(8.11)	3.47[1.03, 11.72]	0.0450	1.63[0.59, 4.53]	0.0866
Important management aspects						
Poor	57(57.89)	8(42.11)	ref		ref	
Good	209(80.08)	52(19.92)	2.92[1.12, 7.63]	0.0285	0.78[0.19, 3.29]	0.7392
Level of Health Facility performance						
Low performance	102(76.12)	32(23.88)	ref			
Good performance	118(80.82)	28(19.18)	1.32[0.75, 2.34]	0.3389		

## Discussion

The purpose of this study was to assess the perceived accountability of HFGCs in primary health care institutions adopting DHFF in Tanzanian municipalities. According to the data, HFGCs members believe that HFGCs have high accountability (78 percent) in DHFF Tanzania’s primary health institutions. These findings are significant and unique because the majority of previous research has focused on assessing social accountability in basic health care [4, 6, 30]. This study was highly precise in examining the accountability of HFGCs under fiscal decentralization in Tanzania, and particularly in underdeveloped nations. The high accountability of HFGCs in the DHFF setting is confirmed by findings in Kenya following the introduction of direct facility financing (DFF) HFGCs, where the ability to fulfill their responsibilities was judged to be satisfactory [7]. In Tanzania, a similar finding was discovered in a study undertaken by Mwakatumbula to analyze the impact of DHFF in primary health facilities, as it was discovered that community autonomy and participation in the management of HFGCs were high in the DHFF setting [31]. Engagement of HFGCs in the planning process of the comprehensive health facility plan and participation in the health facility procurement process has been proven to be significantly associated with HFGC accountability. A similar result was observed in Kenya during the implementation of DFF, where HFGCs participated actively in the planning and budgeting processes [32].

HFGCs, in particular, have been proven to have a high responsibility in areas such as motivating people to join community health funds, financial management, procurement and obtaining medicines and medical commodities. This is the kind of authority that has been devolved to the HFGCs. In certain other developing nations, like Burundi, it was discovered that, despite fiscal autonomy, HFGCs were unable to mobilize facility resources [33]. In other nations, however, fiscal decentralization enabled HFGCs to increase their functioning and responsibilities because they were made responsible for all aspects of service provision, including requesting funds to fund facility operations [34–36].

The participation of HFGCs in resource management at primary health facilities implementing DHFF has been found to be highly associated with their accountability, according to both qualitative and quantitative studies. Respondents mentioned the powers and autonomy afforded by the DHFF system as the reason for their significant engagement [37, 38]. For example, HFGC members have shown through qualitative data that they have been dealing with ensuring financial procedures conform with financial regulations and expenditures based on the budget and facility plan. This finding is consistent with other studies that have been conducted to determine whether the DHFF improved performance in Tanzanian primary health care facilities. It was discovered that community ownership and autonomy have increased to the point where community health structures such as HFGCs are monitoring health service provision [39]. Fiscal decentralization through DFF was found to have strengthened the accountability of HFGCs in financial management in the coast area of Kenya, even though in some other facilities, HFGCs were unable to account for the devolved fiscal powers due to a lack of awareness of their tasks [32, 40].

The inclusion of HFGCs in the procurement process has also been considered to contribute to HFGC accountability. It has been shown that, under DHFF, HFGCs do participate in the entire process of acquiring products such as pharmaceuticals, medical equipment, building materials, and other services necessary by the facilities as outlined in the health facility plan and budget. Indeed, it has been shown that HFGCs are entirely liable for supporting all finances for procurement purposes, as well as ensuring that they see and get what has been acquired. This has boosted transparency in healthcare management. According to a study conducted in India, procurement/logistics is a significant input in the performance of the health system; consequently, when important units such as HFGCs are accountable for the given tasks within the procurement process, successful health care delivery is ensured [34]. However, due to their poor educational level and understanding of health issues, some other members thought that health personnel continue to dominate the procurement process even in their presence. This was also documented in Nepal, where health staff and powerful elites manipulated HFGCs’ participation in the management of health facility operations [41].

Despite HFGC members’ perceptions of high accountability in many accountability indexes, members also view HFGC to have low accountability in managing health professionals and interacting with other stakeholders. The fact that health worker management is still centralized at the council and national levels contributes to HFGCs’ lack of accountability in managing health employees. In the health industry, recruitment, training, and wage payment are not governed by health institutions but rather by the council and the national level. HFGCs are only concerned with complaints involving a specific health worker. However, this should not be used as an excuse by HFGCs because the 2003 health facility guideline states that HFGCs are responsible for supervising facility staff [42]. Furthermore, HFGC’s accountability in communicating with other stakeholders other than the community is minimal. It was anticipated that HFGCs would bring together additional stakeholders such as the private sector, civil societies, and other non-governmental or faith-based organizations to contribute to the establishment of primary health care facilities in their respective areas. However, many HFGCs appear to have focused solely on communication with community members, neglecting other critical issues such as mobilizing stakeholders to deliver health services [30, 43, 44].

The findings have validated the Principal-Agent Theory’s relevance and the responsibility of HFGCs. This is due to the fact that participants in the interviews and focus groups explained that they have been working hard to meet the expectations of their communities (Principal). They also stated that they have used various channels to provide input to the community on what they have been doing at their facilities and how various difficulties have been solved. This is in relation to the principal-agent theory, which states that the agent must account to the principal. The findings revealed that the ability of HFGC members to be elected for another term is contingent on how the HFGC and its members have been carrying out their responsibilities. This is supported by the responses of participants, who agreed in interviews that they served in the HFGC for three terms because community people voted for them based on their performance. As the principal-agent theory explains, this means that communities have been passing judgment (rewarding or penalizing HFGC and their members) after analyzing their functionality in serving communal interests.

In general, this study is very relevant in two aspects for the decentralization of health services and the responsibility of community health systems. First, the study was able to determine the accountability status of HFGCs in primary health care under DHFF, which earlier studies had not done thoroughly. Second, the study revealed elements linked with the accountability of HFGC under DHFF in developing countries that previous studies had not considered. Third, while this study may not have covered all features that can be duplicated in all countries, it has developed an accountability index that may be used to measure the accountability of HFGCs under fiscal decentralization.

## Conclusion

This research provides critical input to policymakers and development partners working to increase the accountability of community health systems in primary health care in developing countries. Fiscal decentralization through DHFF creates a more conducive climate for HFGCs to carry out their delegated obligations, resulting in accountable community health systems. External and internal support are still required to provide a more comfortable/hospitable working environment for health facilities, such as clarifying the duties of HFGCs in managing facility health workers through legal frameworks. There is a need to strengthen HFGCs’ competence to carry out their specific functions, as well as to educate them on the breadth of their powers and autonomy in administering primary health care facilities.

## References

[1] Fetene N, Patel A, Benyam T, Ayde A, Desai MM, Curry L, et al. Experiences of managerial accountability in Ethiopia ‘ s primary healthcare system: a qualitative study. BMC Fam Pract. 2020;1–9. doi: 10.1186/s12875-019-1070-0 33280608 PMC7720402

[2] Belle S Van Mayhew SH. Public accountability needs to be enforced–a case study of the governance arrangements and accountability practices in a rural health district in Ghana. BMC Health Serv Res [Internet]. 2016;1–14. Available from: 10.1186/s12913-016-1836-1 PMC5060000 27729041

[3] Joshi A. Legal Empowerment and Social Accountability: Complementary Strategies Toward Rights-based Development in Health? World Dev [Internet]. 2017;99:160–72. Available from: 10.1016/j.worlddev.2017.07.008

[4] Boydell V, Mcmullen H, Cordero J, Steyn P, Kiare J. Studying social accountability in the context of health system strengthening: innovations and considerations for future work. Heal Res Policy Syst. 2019;1–6. doi: 10.1186/s12961-019-0438-x 30925889 PMC6440124

[5] Lodenstein E, Mafuta E, Kpatchavi AC, Servais J, Dieleman M, Broerse JEW, et al. Social accountability in primary health care in West and Central Africa: exploring the role of health facility committees. BMC Health Serv Res. 2017;1–15. doi: 10.1186/s12913-016-1943-z 28610626 PMC5470232

[6] Lodenstein E, Molenaar JM, Ingemann C, Botha K, Mkandawire JJ, Liem L, et al. “We come as friends”: approaches to social accountability by health committees in Northern Malawi. BMC Health Serv Res [Internet]. 2019;2:1–14. Available from: doi: 10.1186/s12913-019-4069-2 31046748 PMC6498677

[7] Tsofa B, Molyneux S, Gilson L, Goodman C. How does decentralisation affect health sector planning and financial management? a case study of early effects of devolution in Kilifi County, Kenya Lucy Gilson. Int J Equity Health. 2017;16(1):1–12. doi: 10.1186/s12939-017-0649-0 28911325 PMC5599897

[8] Waweru E, Opwora A, Toda M, Fegan G, Edwards T, Goodman C, et al. Are Health Facility Management Committees in Kenya ready to implement financial management tasks Findings from a nationally representative survey. BMC Health Serv Res [Internet]. 2013;13(1):1. Available from: BMC Health Services Research doi: 10.1186/1472-6963-13-404 24107094 PMC3853226

[9] Bovens M. Analysing and Assessing Accountability: A Conceptual Framework 1. Am Polit Sci Rev. 2007;13(4):447–68.

[10] Bakalikwira L, Bananuka J, Kigongo TK, Mukyala V. Accountability in the public health care systems: A developing economy perspective Accountability in the public health care systems: A developing economy perspective. 2017;1975.

[11] Emanuel EJ, Emanuel LL. What Is Accountability in Health Care? Med Public Issues. 2018;Preprint.

[12] Brinkerhoff DW. Accountability and health systems: toward conceptual clarity. Health Policy Plan. 2004;19(6):371–9. doi: 10.1093/heapol/czh052 15459162

[13] Kessy FL. Improving health services through empowered community health governance structures in Tanzania. J Rural Community Dev [Internet]. 2014;9(2):14–31. Available from: http://www.jrcd.ca/viewarticle.php?id=942&layout=abstract

[14] Meyer E, Thomas L, Smith S, Scheepers C. South African health decentralisation: requiring contextually intelligent leaders. Emerald Emerg Mark Case Stud. 2017;7(4):1–23.

[15] Blair H. Citizen Participation and Political Accountabilityfor Public Service Delivery in India: Remapping the World Bank ‘ s Routes. J South Asian Dev. 2018;13(1):54–81.

[16] Kesale AM. Selected Experiences of the Use of the Village Assembly in the Governance at the Grassroots Levels in Ludewa District Council in Tanzania. J Public Adm Gov. 2017;7(2):1–11.

[17] Buchanan A. PRINCIPAL / AGENT THEORY AND DECISIONMAKING I N HEALTH CARE ALLEN B U C H A N A N. Bioethics. 1976;2(4):318–33.

[18] Gailmard S. Accountability and Principal-Agent. OXFORD Univ Press. 2012;(August):1–27.

[19] Kapologwe NA, Kalolo A, Kibusi SM, Chaula Z, Nswilla A, Teuscher T, et al. Understanding the implementation of Direct Health Facility Financing and its effect on health system performance in Tanzania: A non-controlled before and after mixed method study protocol. Heal Res Policy Syst. 2019;17(1):1–13. 10.1186/s12961-018-0400-3 PMC6354343 30700308

[20] Kesale AM. Decentralization by Devolution; Perceptions of Councilors on the Level of their Decision Making Authority in Local Government Experience from Tarime Town Council. J Public Adm Gov. 2016;6(4):1.

[21] Roman TE, Cleary S, McIntyre D. Exploring the functioning of decision space: A review of the available health systems literature. Int J Heal Policy Manag [Internet]. 2017;6(7):365–76. Available from: doi: 10.15171/ijhpm.2017.26 28812832 PMC5505106

[22] Tanzania J ya M wa. MWONGOZO WA UUNDAJI NA UENDESHAJI WA BODI ZA HUDUMA ZA AFYA ZA HALMASHAURI NA KAMATI ZA USIMAMIZI ZA HOSPITALI, VITUO VYA AFYA NA ZAHANATI NCHINI TANZANIA Aprili,a. 2013;

[23] Boex J, Fuller L, Malik A. Decentralized Local Health Services in Tanzania. Urban Inst. 2015;(April).

[24] WHO. Fiduciary Performance and Significant Fiduciary Risks Financial Management Risk C. Report. 2015;(March).

[25] Frederick J Gravetter L-ABF. Research Methods for Behavioral Sciences. 5th Editio. 2012. doi: 10.3390/bs2010001 25379212 PMC4217578

[26] Yahya, Mohamed M. Comment Raising a mirror to quality of care in Tanzania: the five-star assessment. Lancet Glob Heal Comm. 2018;1155–7. doi: 10.1016/S2214-109X(18)30348-6 30196094

[27] Buddhakulsomsiri J, Parthanadee P. Stratified random sampling for estimating billing accuracy in health care systems. Heal Care Manag. 2008;41–54. doi: 10.1007/s10729-007-9023-x 18390167

[28] Israel GD. Determining Sample Size 1. Univ Florida, IFAS Ext. 2012;1–5.

[29] Pandey R, Verma MR, Vulnerability C. SAMPLES ALLOCATION IN DIFFERENT STRATA FOR IMPACT EVALUATION OF. 2008;(January).

[30] Masefield SC, Msosa A, Grugel J. Challenges to effective governance in a low income healthcare system: a qualitative study of stakeholder perceptions in Malawi. BMC Health Serv Res. 2020;20(1):1–16. 10.1186/s12913-020-06002-x PMC7734892 33317520

[31] Mwakatumbula H. The Implementation of Direct Health Facility Financing (DHFF): Prospects and Challenges. Repoa Br. 2021;PB 16(May).

[32] Opwora A, Kabare M, Molyneux S, Goodman C. Direct facility funding as a response to user fee reduction: Implementation and perceived impact among Kenyan health centres and dispensaries. Health Policy Plan. 2010;25(5):406–18. doi: 10.1093/heapol/czq009 20211967 PMC2929466

[33] Falisse JB, Meessen B, Ndayishimiye J, Bossuyt M. Community participation and voice mechanisms under performance-based financing schemes in Burundi. Trop Med Int Heal. 2012;17(5):674–82. doi: 10.1111/j.1365-3156.2012.02973.x 22487362

[34] Panda B, Thakur HP. Decentralization and health system performance—a focused review of dimensions, difficulties, and derivatives in India. BMC Health Serv Res [Internet]. 2016;16(Suppl 6):1–14. Available from: 10.1186/s12913-016-1784-9 PMC5103245 28185593

[35] Jiménez-rubio D. Is fiscal decentralization good for your health? Evidence from a panel of OECD Is fiscal decentralization good for your health? Evidence from a panel of OECD countries Dolores Jiménez-Rubio. 2014;(December 2010).

[36] Samadi AH, Keshtkaran A, Kavosi Z, Vahedi S. The effect of fiscal decentralization on under-five mortality in Iran: A panel data analysis. Int J Heal Policy Manag. 2013;1(4):301–6. doi: 10.15171/ijhpm.2013.60 24596888 PMC3937898

[37] Martinez-Vazquez J. The Impact of Fiscal Decentralization. Issues in Theory and Challenges in Practice. Adb. 2011;1–27.

[38] The World Bank. Fiduciary Systems Assessment Tanzania–Strengthening Primary Health Care Services for Results. 2015;(March).

[39] Kajuni BA, Mpenzi DF. The Direct Health Facility Financing Impact on Financial Management in Kaliua District Council, Tabora Tanzania. World J Bus Manag. 2021;7(1):1.

[40] Goodman A. O, K M., M S. Health facility committees and facility management—exploring the nature and depth of their roles in Coast Province, Kenya. BMC Health Serv Res [Internet]. 2011;11:229. Available from: http://www.embase.com/search/results?subaction=viewrecord&from=export&id=L560043607 doi: 10.1186/1472-6963-11-229 21936958 PMC3197493

[41] Gurung G, Derrett S, Hill PC, Gauld R. Nepal’s Health Facility Operation and Management Committees: Exploring community participation and influence in the Dang district’s primary care clinics. Prim Heal Care Res Dev. 2018;19(5):492–502. 10.1017/S1463423618000026 PMC6452933 29374506

[42] Maluka SO. Strengthening fairness, transparency and accountability in health care priority setting at district level in Tanzania. Glob Health Action. 2011;4. doi: 10.3402/gha.v4i0.7829 22072991 PMC3211296

[43] Coy D, Dixon K. The public accountability index: crafting a parametric disclosure index for annual reports. 2004;36:79–106.

[44] Liwanag HJ, Wyss K. Optimising decentralisation for the health sector by exploring the synergy of decision space, capacity and accountability: Insights from the Philippines. Heal Res Policy Syst. 2019;17(1):1–16. doi: 10.1186/s12961-018-0402-1 30630469 PMC6327786

